# One-Step Low Temperature Hydrothermal Synthesis of Flexible TiO_2_/PVDF@MoS_2_ Core-Shell Heterostructured Fibers for Visible-Light-Driven Photocatalysis and Self-Cleaning

**DOI:** 10.3390/nano9030431

**Published:** 2019-03-14

**Authors:** Zhi-Guang Zhang, Hui Liu, Xiao-Xiong Wang, Jun Zhang, Miao Yu, Seeram Ramakrishna, Yun-Ze Long

**Affiliations:** 1 Collaborative Innovation Center for Nanomaterials & Devices, College of Physics, Qingdao University, Qingdao 266071, China; zhangzhiguangphysics@126.com (Z.-G.Z.); lhqddx@163.com (H.L.); wangxiaoxiong69@163.com (X.-X.W.); iamjunzhang@163.com (J.Z.); my2373@columbia.edu (M.Y.); 2 College of Science & Information, Qingdao Agricultural University, Qingdao 266109, China; 3 Department of Mechanical Engineering, Columbia University, New York, NY 10027, USA; 4 Center for Nanofibers & Nanotechnology, Nanoscience & Nanotechnology Initiative, Faculty of Engineering, National University of Singapore, Singapore 117576, Singapore; seeram@nus.edu.sg

**Keywords:** low temperature, core-shell heterostructure, visible light, photocatalysis, self-cleaning

## Abstract

Novel flexible and recyclable core-shell heterostructured fibers based on cauliflower-like MoS_2_ and TiO_2_/PVDF fibers have been designed through one-step hydrothermal treatment based on electrospun tetrabutyl orthotitanate (TBOT)/PVDF fibers. The low hydrothermal temperature avoids the high temperature process and keeps the flexibility of the as-synthesized materials. The formation mechanism of the resultant product is discussed in detail. The composite of MoS_2_ not only expands the light harvesting window to include visible light, but also increases the separation efficiency of photo-generated electrons and holes. The as-prepared product has proven to possess excellent and stable photocatalytic activity in the degradation of Rhodamine B and levofloxacin hydrochloride under visible light irradiation. In addition, the TiO_2_/PVDF@MoS_2_ core-shell heterostructured fibers exhibit self-cleaning property to dye droplets under visible light irradiation. Meanwhile, due to its hydrophobicity, the resultant product can automatically remove dust on its surface under the rolling condition of droplets. Hence, the as-prepared product cannot only degrade the contaminated compounds on the surface of the material, but also reduce the maintenance cost of the material due to its self-cleaning performance. Therefore, the as-prepared product possesses potential applications in degradation of organic pollutants and water treatment, which makes it a prospective material in the field of environmental treatment.

## 1. Introduction

As a highly efficient, economical and environmentally friendly “green” technology, photocatalysis offers tremendous potential for environmental protection and energy conversion. Therefore, the production of advanced photocatalytic materials is one of the main strategies to solve the current global environmental needs [1,2]. Titanium dioxide (TiO_2_) has proven to be a promising candidate for photocatalysts in various transition metal oxide semiconductors over the past few decades due to its good physicochemical properties, non-toxicity, low-cost, stable chemical and photonic properties [3]. However, as an n-type wide-bandgap semiconductor, TiO_2_ absorbs only ultraviolet light, which accounts for only 4% of total sunlight in the solar spectrum. In addition, the recombination rate of photo-generated electron hole pairs in TiO_2_ is high, resulting in low quantum efficiency and low photocatalytic activity. Furthermore, for the traditional powder-like photocatalyst, it is also very difficult to separate and recover a photocatalyst from the reaction solution after the photocatalytic reaction. 

Therefore, the key for preparation of a highly active and recyclable TiO_2_-based photocatalyst is to find a strategy for inhibiting photo-generated electron-hole recombination, narrowing the band gap and easy recovery and recycle. To this end, many strategies have been developed, one of which is the composition of narrow band gap semiconductors to TiO_2_ to form a heterogeneous structure [4,5,6]. On the one hand, the composition of narrow band gap semiconductors can improve the photocatalytic activity of TiO_2_-based photocatalysts by expanding their light capture window to the visible range [7]. On the other hand, the heterostructure between narrow band gap semiconductors and TiO_2_ can also enhance the charge separation by coupling two semiconductor structures with matched energy levels, thereby increasing the photocatalytic activity and efficiency of the TiO_2_-based photocatalyst [8,9]. Among many narrow bandgap semiconductors, molybdenum disulfide (MoS_2_) has become the material of choice for composite heterostructures due to its large reserves, low cost, and excellent electronic and optical properties [10,11,12]. Zheng et al. synthesized hierarchical MoS_2_ nanosheet@TiO_2_ nanotube arrays by combining the anodic oxidation method and hydrothermal method [13]. The as-prepared hierarchical composite materials are of enhanced photocatalytic and photocurrent performances. Liu et al. prepared 3D sandwich-like heterojunction structured mesoporous black TiO_2_/MoS_2_/TiO_2_ nanosheets which have 89.86% methyl orange degradation rate and 0.56 mmol·h^−1^·g^−1^ hydrogen production rate [14]. However, the above-mentioned several kinds of composite photocatalysts still exist in the form of powder. Therefore, there is still a disadvantage for recovery and recycle when the photocatalyst was used in a reaction solution. In order to improve the recovery and recycle of a photocatalyst, the method of supporting the photocatalytic material on an inorganic porous material or a polymer material has been reported [15,16,17,18]. Zhang et al. fabricated 3D MoS_2_ nanosheet/TiO_2_ nanofiber heterostructures by using the electrospinning method combined with hydrothermal treatment [19]. The as-synthesized fibers presented enhanced performance in the photocatalytic decomposition of organic dyes under UV light irradiation. However, a high temperature post-sintering process was employed in the synthesis procedure to remove the polymer composition, which resulted in the as-synthesized nanofibers being too fragile and difficult to reuse [20]. In order to solve the problem of difficult to recover photocatalysts, flexible substrate materials have been introduced as photocatalyst carriers [21,22,23,24]. Lin et al. synthesized a novel floating sheet used in solar photocatalytic water splitting. The as-synthesized novel floating sheet consists of WSe_2_ film laser-deposited on a carbon foam substrate and nanodiamond-embedded Cu_2_O photocatalysts, which has better reusability [21]. Yu et al. synthesized AgX (X = Br, I)-TiO_2_ nanoparticles immobilized on polyacrylonitrile (PAN) nanofibers by combining the electrospinning technique, solvothermal synthesis, physical adsorption, and gas/solid reaction. The as-prepared composite showed excellent visible light catalytic performance against various pollutants [24]. Polyvinylidene difluoride (PVDF), a widely used commercial polymer material, which has the advantages of good thermal stability, high mechanical strength, and chemical resistance, is very suitable as a carrier for flexible composite materials [25]. Our previous work showed that the use of PVDF as photocatalyst carrier can be a good solution for the separation and recovery of photocatalysts from the reaction solution [26]. 

In this paper, novel flexible, recyclable, and reusable TiO_2_/PVDF@MoS_2_ core-shell heterostructured fibers were synthesized by one-step hydrothermal treatment at low temperature based on electrospun tetrabutyl orthotitanate (TBOT)/PVDF fibers. The one-step hydrothermal method is very simple and feasible. In addition, the low hydrothermal temperature, avoiding the high temperature process, will not damage the flexibility of the as-synthesized materials. A large number of cauliflower-like MoS_2_ nanoparticles were grown on the surface of TiO_2_/PVDF fibers forming a flexible core-shell heterostructure. The as-prepared materials have good flexibility, recyclability, and reusable property. The mechanism that the flexible TiO_2_/PVDF@MoS_2_ core-shell heterostructured fibers have excellent photocatalytic activity on organic pollutants under visible light was discussed. The self-cleaning properties of the resultant product were also investigated. The results show that the photocatalytic activity of TiO_2_ crystal is significantly increased in the presence of MoS_2_ as a co-catalyst. Furthermore, the as-prepared product cannot only degrade the contaminated compounds on the surface of the material, but also reduce the maintenance cost of the material due to its self-cleaning performance. Therefore, the application of flexible TiO_2_/PVDF@MoS_2_ core-shell heterostructured fibers to the decomposition of toxic and harmful organic pollutants is of great significance for environmental protection.

## 2. Experimental

### 2.1. Materials

PVDF (FR904) was purchased from Shanghai 3F New Materials Co., Ltd. (Shanghai, China), Degussa P25 (80% anatase and 20% rutile) was purchased from Evonik Degussa Company (Shanghai, China), *N*,*N*-dimethylformamide (DMF, AR, 99.5%), acetone (CP, 99.0%), sulphuric acid (H_2_SO_4_, CP, 95.0%~98.0%), TBOT(CP, 98.0%), Sodium molybdate dihydrate (Na_2_MoO_4_·2H_2_O, AR, 99.0%) and thiourea (AR, 99.0%) were purchased from Sinopharm Chemical Reagent Co., Ltd. (Shanghai, China). All reagents were used as received without any further purification.

### 2.2. Preparation of TBOT/PVDF Fibers

A 4.0 g sample of PVDF powder was added into a mixture of solvents with 10 g DMF and 10 g acetone and stirred vigorously at 40 °C until the solution was clear and transparent. Then, 10 mL of TBOT was added to the clear solution and stirring was continued for 1 h at 40 °C. Electrospinning was operated using a 5 mL syringe containing the TBOT/PVDF precursor solution with a blunt metal needle. The fiber collector was a stainless steel roller wrapped with a sheet of aluminum foil and operating a rotation speed of about 250 rpm. A DC voltage supply with a setting of 9 kV was placed between the needle tip and the collector with the needle tip and collector at a distance of 11 cm. The fibrous mats collected on the aluminium foils were dried at 60 °C for 10 h after spinning to eliminate any remaining solvent. All the as-prepared TBOT/PVDF fibers were cut into small pieces of 2.5 cm × 2.5 cm for the hydrothermal treatment.

### 2.3. Fabrication of TiO_2_/PVDF@MoS_2_ Core-Shell Heterostructured Fibers

0.5 mmol Na_2_MoO_4_·2H_2_O and 2.5 mmol thiourea were added into 30 mL of 0.5 M sulfuric acid solution and stirred for 30 min. The solution was then transferred to a 50 mL stainless steel autoclave. The TBOT/PVDF small pieces were then placed into the stainless steel autoclave. The hydrothermal reaction was carried out at 150 °C for 24 h, and then the flexible TiO_2_/PVDF@MoS_2_ core-shell heterostructured fibers were obtained. For comparison, the same conditions were carried out without Mo source and S source to form TiO_2_/PVDF fibrous mat, and the same conditions using PVDF fibers mat to form MoS_2_/PVDF fibrous mat. The as-obtained fibers mats were thoroughly washed with ethyl alcohol and deionized water, followed by drying in air at 60 °C for 10 h after the hydrothermal treatment. 

### 2.4. Characterization

X-ray diffraction (XRD) patterns were taken with a Rigaku SmartLab X-ray diffractometer (Rigaku, Tokyo, Japan) using a Cu-Kα radiation in the 2θ range of 10–80° at room temperature. The scanning electron microscopy (SEM) images of the as-synthesized samples were taken using a JEOL JSM-7800F field emission scanning electron microscope (JEOL, Tokyo, Japan). A JEOL JEM-2100Plus transmission electron microscopy (TEM) (JEOL, Tokyo, Japan) attached energy dispersive spectroscopy (EDS) was used for observation of the as-obtained samples. X-ray photoelectron spectroscopy (XPS) measurements were carried out on a Thermo Scientific Escalab 250Xi system (Thermo Scientific, Shanghai, China) with an Al Kα X-ray source. The Brunauer-Emmett-Teller (BET) specific surface area was performed on a Quantachrome Autosorb-IQ-MP/XR nitrogen adsorption apparatus (Quantachrome, Shanghai, China). UV-Vis diffuse reflectance spectra (DRS) of the as-prepared samples were taken using a Shimadzu UV-2600 spectrophotometer (Shimadzu, Tokyo, Japan) with an integrating sphere accessory, in which BaSO_4_ was utilized as a diffuse reflectance standard. A Hitachi F-2500 fluorescence spectrometer (Hitachi, Tokyo, Japan) with a Xe lamp was used to determine the photoluminescence spectra (PL) of the resultant membranes using 320 nm as the excitation wavelength at room temperature. 

### 2.5. Photocatalytic Activity

The photocatalytic performances of the as-synthesized samples were evaluated by decomposing the model pollutants rhodamineB (RhB, 15 mg·L^−1^) and levofloxacin hydrochloride (LVFX, 5 mg·L^−1^) under visible-light irradiation at room temperature. In order to compare the photocatalytic ability to degrade dye contaminants, Degussa P25 was purchased and applied to the degradation of RhB photocatalytic experiments. In photodegradation experiments, the photocatalysts (1 g·L^−1^) were put into a 100 mL quartz tube with 60 mL target pollutants solution and magnetically stirred in the dark for 45 min to ensure the adsorption-desorption equilibrium of target pollutants on the catalysts surface. Then this system was placed under a 9 W white light LED (Figure S1, Supporting Information (SI)) with a distance of 4.0 cm apart away from the quartz tube where the power density of the white LED lamp is 0.9 mW·cm^−2^. At selected time intervals, 3 mL of aliquots were collected and centrifuged to remove the particles, then sampled to analyze the concentration of RhB remaining in the solution by measuring its absorbance at 554 nm for RhB (292 nm for LVFX) using a Shimadzu UV-2600 spectrophotometer. For the membranes reaction system, the analyzed aliquot was quickly poured back into the quartz tube to ensure a roughly equivalent volume of solution after every assay. The photodegradation efficiency was expressed as C/C_0_, where C is the absorption of RhB absorption spectrum at 554 nm (292 nm for LVFX) at selected time intervals and C_0_ is the absorption of the starting concentration. In order to investigate the recycle stability, the as-prepared TiO_2_/PVDF@MoS_2_ core-shell heterostructured fibers were washed with ethyl alcohol and deionized water, and then dried in air for the next photodegradation process.

Control experiments on the photodegradation of RhB were carried out by using ethylene diamine tetraacetic acid (EDTA, 10 mM), tertiary butanol (tBuOH, 10 mM) and nitrogen (N_2_) as the photo-generated holes (h^+^), the hydroxyl radicals (OH^•^) and the superoxide anion radicals ($O_{2}^{-•}$) scavenger, respectively.

### 2.6. Self-Cleaning Performance

#### 2.6.1. Hydrophobicity Property

The hydrophobicity of the TiO_2_/PVDF@MoS_2_ core-shell heterostructured fibers is evaluated by measuring the contact angle of the droplets (including H_2_O, RhB, methylene blue (MB)) on the material under ambient temperature. The water contact angle measurements were carried out by the drop method on a Theta Attension optical contact angle instrument (Biolin Scientific, Stockholm, Sweden). 

#### 2.6.2. Fading of Dye Droplets

The RhB and MB dye droplets with a concentration of 10 mg·L^−1^ were dripped onto the TiO_2_/PVDF@MoS_2_ core-shell heterostructured fibers and irradiated under visible light. An optical photograph was taken every 25 min to compare the color of the dye, which was used to characterize the self-cleaning performance of the as-prepared product to the surface colored pollutants.

#### 2.6.3. Removal of Dust on Sample Surface

In order to evaluate the self-cleaning effect of the TiO_2_/PVDF@MoS_2_ core-shell heterostructured fibers on the dust on its surface, the dust was scattered on the surface of the sample before measurement. Then, a drop of water was dropped on the surface of the sample. Tilted the sample slightly to make the droplet move on the sample surface and take away the dust, thus making the material surface clean. 

## 3. Results and Discussion

### 3.1. Synthesis and Application Process

**Figure 1:**
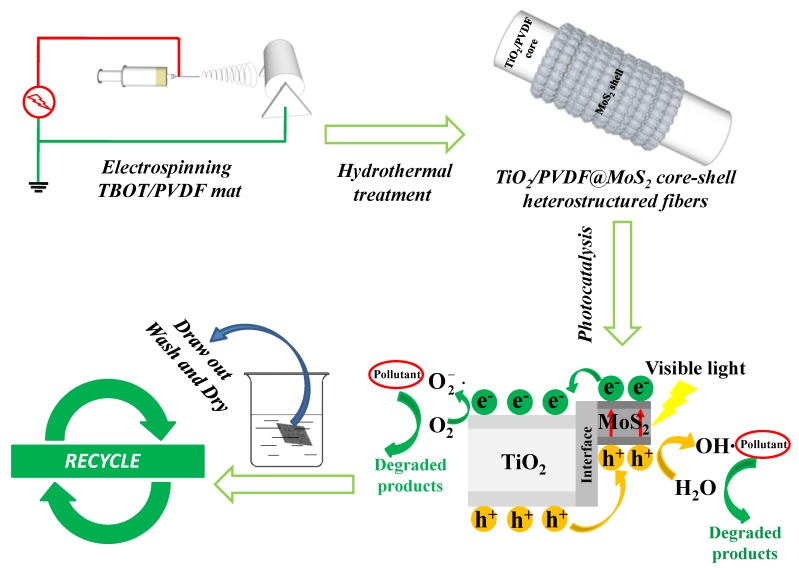
Schematic illustration for the synthesis and application process of TiO_2_/PVDF@MoS_2_ core-shell heterostructured fibers.

A brief synthesis and application process of TiO_2_/PVDF@MoS_2_ core-shell heterostructured fibers are depicted in Figure 1. Firstly, the TBOT/PVDF fibrous mat was obtained by electrospinning TBOT/PVDF homogeneous solution. Secondly, the as-prepared TBOT/PVDF fibrous mat was cut into small pieces, followed by hydrothermal treatment to synthesize flexible TiO_2_/PVDF@MoS_2_ core-shell heterostructured fibers. Thirdly, the flexible TiO_2_/PVDF@MoS_2_ core-shell heterostructured fibers were applied to degrade organic pollutant under visible light. Finally, the flexible TiO_2_/PVDF@MoS_2_ core-shell heterostructured fibers were drawn out from the reaction system and thoroughly washed with ethyl alcohol and deionized water, followed by drying in air at 60 °C for 10 h for the next photocatalytic experiment.

### 3.2. Structure and Morphology Characteristics

**Figure 2:**
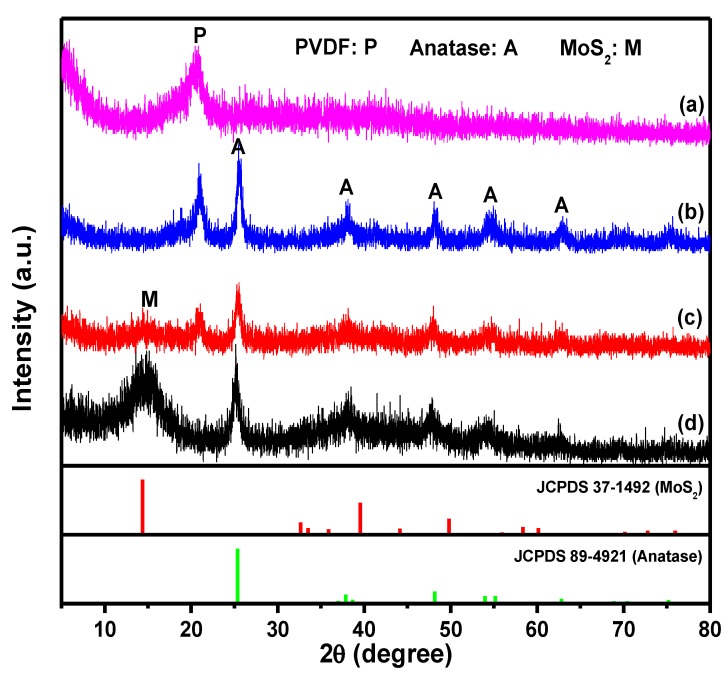
XRD patterns of (**a**) TBOT/PVDF fibers, (**b**) TiO_2_/PVDF hybrid fibers, (**c**) TiO_2_/PVDF@MoS_2_ core-shell heterostructured fibers, and (**d**) MoS_2_ and TiO_2_ powders.

Figure 2 depicts the X-ray diffraction (XRD) patterns of the as-prepared samples. As displayed in Figure 2 curve (a), no obvious diffraction peak was detected except the diffraction peak 2θ at 20.7° which can be assigned to the β phase of PVDF [27,28]. This means that the TiO_2_ small crystal in the TBOT/PVDF hybrid fibers mat, formed through TBOT hydrolyzed with the H_2_O in air, was mainly in the amorphous structure. After hydrothermal reaction in 0.5 M H_2_SO_4_ at 150 °C for 24 h, the characteristic diffraction peaks of anatase phase TiO_2_ (PDF card 89-4921, Joint Committee on Powder Diffraction Standards (JCPDS)) appeared in the prepared hybrid material, whose diffraction peaks 2θ at 25.6, 37.9, 48.2, 54.4, and 62.8° as shown in the curve (b). The addition of H_2_SO_4_ ensured the formation of anatase phase TiO_2_ [29]. In addition to the above-mentioned anatase phase TiO_2_ diffraction peaks in the curve (b), a weak and broad diffraction peak at 14.4° can be detected in the curve (c), which can be indexed to the (002) crystal face of MoS_2_ (PDF card 37-1492, JCPDS). Comparing curve (c) with curve (a) and (b), it can be seen that the intensity of the diffraction peaks of PVDF and anatase TiO_2_ decreased after synthesizing MoS_2_ on TiO_2_/PVDF fibers, indicating that the surface area of TiO_2_/PVDF was covered with MoS_2_ that forms a core-shell structure. In order to further determine whether the preparation of MoS_2_ was successful, the powder in the hydrothermal autoclave was centrifuged after hydrothermal reaction and tested (shown in curve (d)). Comparing curves (d) with (c), it was obvious that all the diffraction peaks were at the same position except the diffraction peaks of PVDF especially, where the diffraction peak of MoS_2_ became stronger, indicating the MoS_2_ was synthesized successfully. As a comparison, the XRD patterns of pure PVDF fibers mat, MoS_2_/PVDF hybrid fibers mat and MoS_2_ powder remaining in the hydrothermal autoclave after hydrothermal synthesizing MoS_2_/PVDF hybrid fibers mat were displayed in Figure S2 (SI). It can be concluded the MoS_2_ was successfully grown on the PVDF fibers.

**Figure 3:**
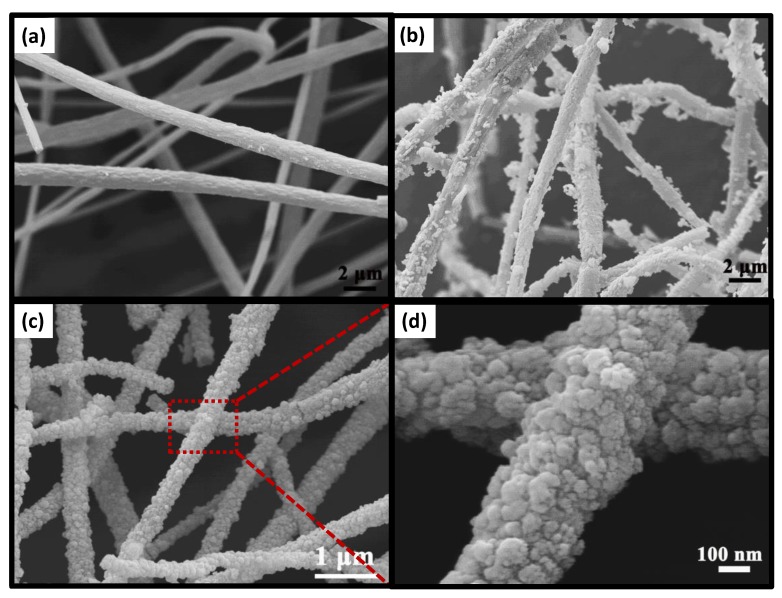
SEM images of (**a**) TBOT/PVDF fibers, (**b**) TiO_2_/PVDF fibers, (**c**) TiO_2_/PVDF@MoS_2_ fibers, and (**d**) high resolution SEM image of TiO_2_/PVDF@MoS_2_ core-shell heterostructured fibers.

Figure 3 shows the typical SEM images of TBOT/PVDF fibers, TiO_2_/PVDF fibers and TiO_2_/PVDF@MoS_2_ core-shell heterostructured fibers, respectively. As illustrated in Figure 3a, TBOT/PVDF fibers randomly distributed with a rough surface different from pure PVDF fibers synthesized in our previous work [26], mainly due to the TBOT component in the fibers hydrolyzed with the H_2_O in the atmosphere. It can be seen from Figure 3a that the fiber diameter distribution was very uneven, some particularly thick and some particularly fine. Besides, it is obvious that some fibers were broken, and the same phenomenon also appeared in Figure 3b,c. Considering the blend of PVDF polymer and TBOT in the precursor solution, the spinning needle would be blocked, caused by the hydrolysis reaction between TBOT and H_2_O in the air during the electrospinning process, which would lead to the diameter distribution of fibers nonuniform. While the TBOT component in the fibers continuing to hydrolyze with H_2_O in the air will cause fiber fracture. After being hydrolyzed in 0.5 M H_2_SO_4_ at 150 °C for 24 h, anatase phase TiO_2_ occurred on the fiber surface (shown in Figure 3b), which can be confirmed by the XRD patterns. As depicted in Figure 3b, anatase phase TiO_2_ particles with irregular shapes and sizes were randomly distributed on the fibers surface. During hydrothermal growth process, a portion of the TBOT component in the fibers dissolved in the reaction liquid while the other part remained in the fibers. In an acid solution environment, both of them began to hydrolyze to form TiO_2_ at the same time. Due to the lack of nucleation centers in the liquid, a part of the TBOT dissolved in the liquid hydrolyzed and grew homogeneously to form TiO_2_ powder directly, and the other part combined with the TiO_2_ particles formed by TBOT hydrolysis in the fiber to become larger particles. As a result, morphology as shown in Figure 3b was formed.

As presented in Figure 3c,d, unlike the common layered structure of MoS_2_, a large number of cauliflower MoS_2_ particles appeared on the fibers surface forming core-shell structure. In contrast to the morphology of the TiO_2_ particles in Figure 3b, these cauliflower MoS_2_ particles were relatively uniform and dense. For TiO_2_/PVDF@MoS_2_ fibers, the hydrolysis of TBOT and the growth of MoS_2_ were carried out simultaneously. Since there were many MoS_2_ particles in the solution, this makes the TiO_2_ in the liquid hydrolyzed by TBOT more likely to combine with the MoS_2_ to form heterogeneous growth rather than to grow on the fibers. Therefore, no particularly large TiO_2_ particles were formed on the fibers. The amount of Na_2_MoO_4_ and (H_2_N)_2_CS in the solution was sufficient so that MoS_2_ can either directly form a powder in solution or grow on the surface of the TiO_2_/PVDF core, which was the main reason for the difference in topography between Figure 3b,c.

**Figure 4:**
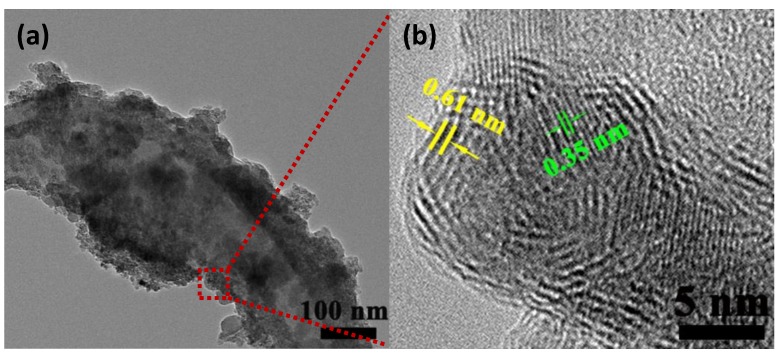
TEM image of TiO_2_/PVDF@MoS_2_ core-shell heterostructured fiber (**a**) and high-resolution TEM image of TiO_2_/PVDF@MoS_2_ core-shell heterostructured fiber (**b**).

The morphology of TiO_2_/PVDF@MoS_2_ core-shell heterostructured fiber was further confirmed by TEM and HRTEM, as shown in Figure 4. It is clearly observed that the very fine MoS_2_ particle grew on the surface of TiO_2_/PVDF fiber and core–shell structures appeared, as displayed in Figure 4a. The high-resolution TEM image showed that the MoS_2_ particles with several layer thicknesses were about 5 nm in size. In addition, the MoS_2_ particles with an average spacing of 0.61 nm can be seen, which belongs to the (002) facet of MoS_2_ [30,31,32]. In Figure 4b, the lattice spacing of TiO_2_ was measured to be 0.35 nm, which was in close agreement with (101) facets of anatase TiO_2_ [33]. The lattice spacing shown in the HRTEM images is consistent with the XRD results, further confirming the formation of TiO_2_/PVDF@MoS_2_ core-shell structure.

The EDS technique was employed for further detecting the elemental composition of as-prepared TiO_2_/PVDF@MoS_2_ fiber. The EDS spectroscopy in Figure S3 (SI) displays the elements of C, O, F, Cu, Ti, S, and Mo, of which Cu was from copper mesh brackets, confirming the presence of PVDF, TiO_2_, and MoS_2_.

**Figure 5:**
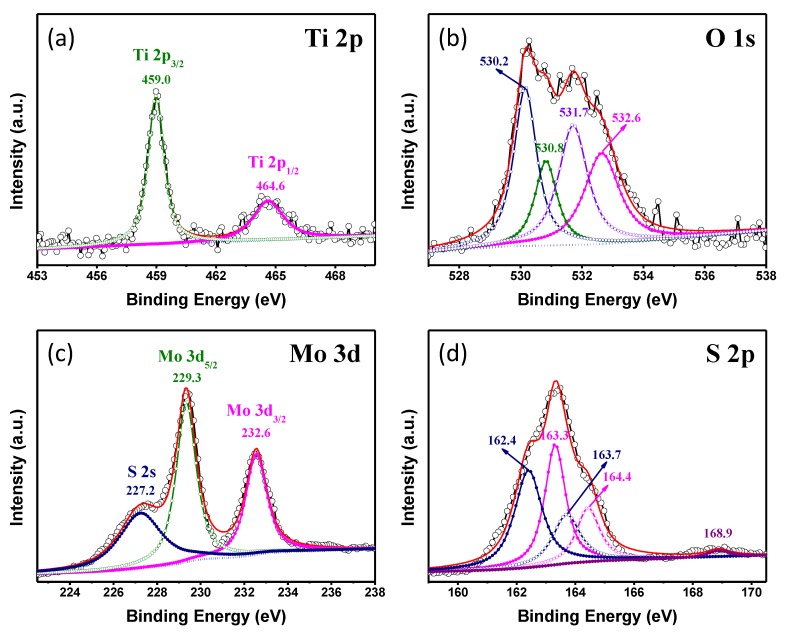
High-resolution XPS spectra of (**a**) Ti 2p, (**b**) O 1s, (**c**) Mo 3d and (**d**) S 2p in TiO_2_/PVDF@MoS_2_ core-shell heterostructured fiber.

The chemical composition information and the bonding configuration of the as-prepared products were determined by XPS analysis. Characteristic peaks from Mo, S, O, Ti, F, and C can be clearly found from the XPS survey spectra, as shown in Figure S4 (SI). Figure 5 illustrates the high-resolution XPS spectra of Ti 2p, O 1s, Mo 3d, and S 2p. As shown in Figure 5a, the binding energies of Ti 2p_3/2_ and Ti 2p_1/2_ peaks were located at 459.0 and 464.6 eV, respectively [34]. The peak of O1s was broken up into four peaks (shown in Figure 5b), respectively, corresponding to Ti-O of TiO_2_ (530.2 eV), Ti-O-Mo bonds between MoS_2_ and TiO_2_ (530.8 eV), hydroxyl group (531.7 eV), and C-O bond (532.6 eV) in the resultant product [35,36,37]. In Mo 3d profiles (Figure 5c), the peaks at 232.6 and 229.3 eV were corresponding to 3d_3/2_ and 3d_5/2_ of Mo^4+^, respectively. And the satellite-peak at 227.2 eV ascribed to 2s of S species [38]. Meanwhile, the peaks of S element could be divided into three different chemical environments, as displayed in Figure 5d. The broad spectra could be fitted with sets of doublets related to spin orbit split to 2p_3/2_ and 2p_1/2_. The peaks at binding energies of 162.4 and 163.7 eV were assigned to the 2p_3/2_ and 2p_1/2_ of S^2−^ [13]. Whereas, the peaks at 163.3 and 164.4 eV may be assigned to C-S bonds or related to the presence of bridging S_2_^2−^ [39,40]. Besides, the weak peak at 168.9 eV was related to the residual of SO_4_^2−^ in the as-prepared sample [41,42]. These results further confirmed the presence of MoS_2_, TiO_2_, and PVDF, which agreed well with the XRD and TEM results.

**Figure 6:**
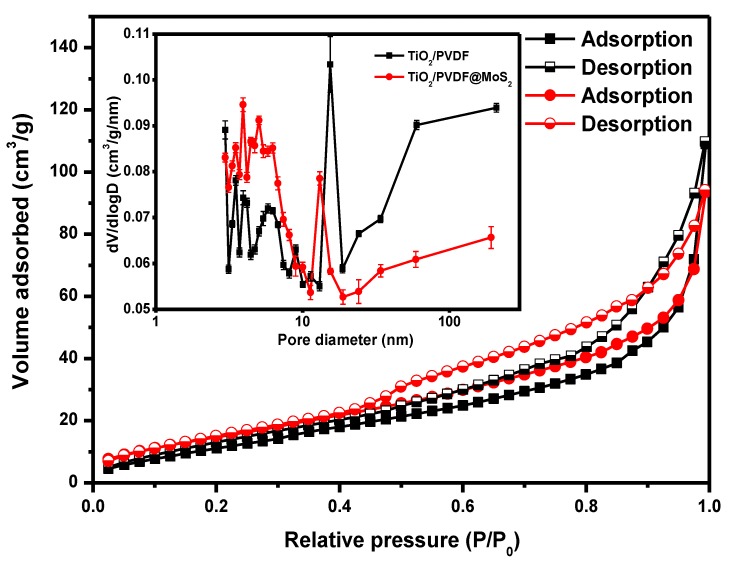
Nitrogen adsorption–desorption isotherms and the corresponding pore-diameter distribution curves (inset) of TiO_2_/PVDF (Black line) and TiO_2_/PVDF@MoS_2_ core-shell heterostructured fibers (Red line).

To investigate the specific surface area of resultant products, nitrogen adsorption-desorption analysis was carried out by using the BET method. Figure 6 displays the nitrogen adsorption–desorption isotherms of TiO_2_/PVDF and TiO_2_/PVDF@MoS_2_ fibers, and the inset illustrates the corresponding pore diameter distribution by using the Barrett-Joyner-Halenda (BJH) method. The isotherm curves of TiO_2_/PVDF and TiO_2_/PVDF@MoS_2_ fibers were well in agreement with the type IV isotherm behavior with H3 hysteresis [43,44].

The surface area of TiO_2_/PVDF fibers was 49.5 m^2^·g^−1^, whereas the TiO_2_/PVDF@MoS_2_ core-shell heterostructured fibers had a surface area of 59.2 m^2^·g^−1^. Meanwhile, the pore diameter distribution of TiO_2_/PVDF and TiO_2_/PVDF@MoS_2_ core-shell heterostructured fibers was very irregular, as shown in the inset of Figure 6. Compared to the TiO_2_/PVDF fibers, the pore diameter distribution of TiO_2_/PVDF@MoS_2_ core-shell heterostructured fibers tended to be smaller, mainly due to the fact that the surface of TiO_2_/PVDF fibers was covered with smaller MoS_2_ particles instead of larger TiO_2_ particles, which was consistent with the SEM results. Hence, the as-obtained TiO_2_/PVDF@MoS_2_ core-shell heterostructured fibers possessed much more absorption interface.

### 3.3. Optical Characteristics

**Figure 7:**
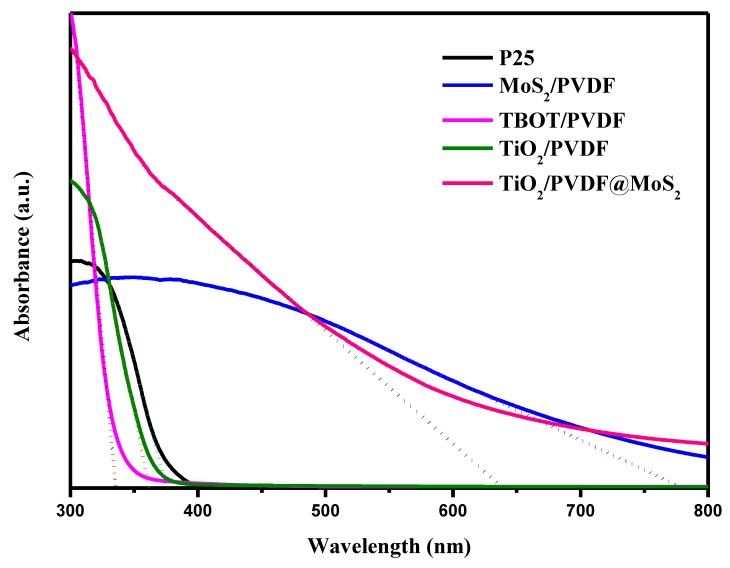
UV-vis diffuses reflectance spectra of different samples: P25, PVDF, MoS_2_/PVDF, TBOT/PVDF, TiO_2_/PVDF and TiO_2_/PVDF@MoS_2_.

Optical absorption of P25, PVDF, MoS_2_/PVDF, TBOT/PVDF, TiO_2_/PVDF, and TiO_2_/PVDF@MoS_2_ were investigated by UV-Vis diffuse reflectance spectra, displayed in Figure 7. It can be found that all materials had a strong absorption except PVDF at wavelengths below 400 nm. Most especially, the TBOT/PVDF fibers had the strongest absorption in the ultraviolet region, owing to formation of amorphous structured TiO_2_ crystal through TBOT hydrolyzed with the H_2_O in air. In the visible light region, the samples with MoS_2_ particles covered on the surface present enhanced absorption character compared to P25 and TiO_2_/PVDF fibers.

For an indirect-band-gap semiconductor, the band-gap energy can be acquired by equation Eg = 1240/λ_g_ (eV), where λ_g_ is the absorption edge calculated from the intercept between the tangent of the absorption curve and the abscissa coordinate [45]. The absorption edge and band-gap energy for P25, TBOT/PVDF, TiO_2_/PVDF, MoS_2_/PVDF, and TiO_2_/PVDF@ MoS_2_ was displayed in Table 1. Obviously, the band-gap energy of the TiO_2_/PVDF@ MoS_2_ was different from the MoS_2_/PVDF and the TiO_2_/PVDF, mainly due to its core-shell structure [46].

Benefitting from the addition of narrow band gap MoS_2_, the absorption edge of TiO_2_/PVDF@ MoS_2_ core-shell heterostructured fibers shifted to longer wavelength (1.9 eV) compared to that of TiO_2_/PVDF fibers at 3.4 eV. Therefore, the core-shell heterostructured TiO_2_/PVDF@ MoS_2_ fibers offered enhanced light harvesting in the visible region of the solar spectrum, and thus, presented considerable photocatalytic abilities under visible light illumination.

**Figure 8:**
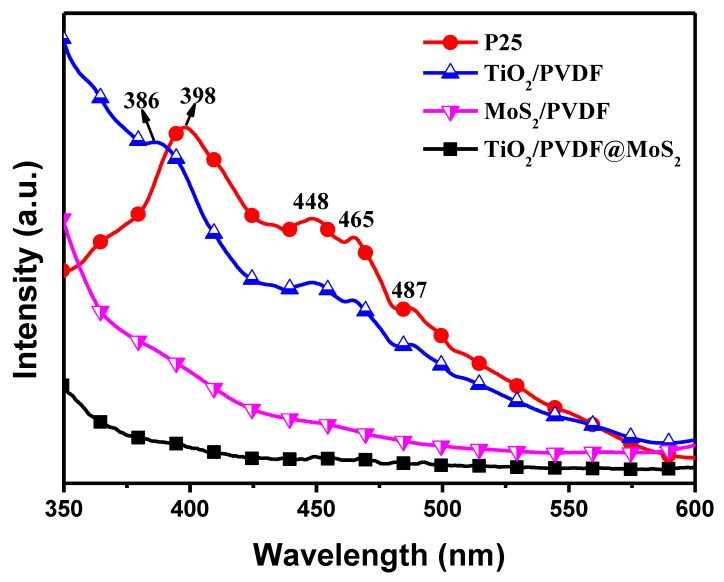
PL spectra of P25, TiO_2_/PVDF, MoS_2_/PVDF and TiO_2_/PVDF@MoS_2_.

The photoluminescence (PL) spectra were usually used to evaluate the efficiency of charge trapping and recombination of photo-induced electron-hole pairs in the semiconductor [47,48]. Figure 8 shows the PL spectra of P25, TiO_2_/PVDF, MoS_2_/PVDF, and TiO_2_/PVDF@MoS_2_. There were four main emission peaks for P25 and TiO_2_/PVDF, respectively. The peak located at 398 nm (≈3.12 eV) belonged to P25; meanwhile, the peaks located at 386 nm (≈3.21 eV) belonged to TiO_2_/PVDF. The other three peaks, located at 448 nm (≈2.77 eV), 465 nm (2.67 eV) and 487 nm (≈2.55 eV), respectively, appear in both P25 and TiO_2_/PVDF. The first peak of these two materials corresponded to their near-band gap emission [26], whereas the other three peaks were likely assigned to the emission of oxygen vacancies related defect formed in the synthetic process [49,50,51]. Besides, it is obviously that P25 has the highest PL intensity, which means having the highest photo-generated electron-hole recombination. Interestingly, the PL intensity of TiO_2_/PVDF fibers was weaker than that of P25, implying a relatively higher photo-generated electron-hole separation efficiency. The formation of F-Ti coordination bond in the fibers and the relatively high ionic conductivity of PVDF as a ferroelectric material should take the main responsibility [22]. In addition, the TiO_2_/PVDF@MoS_2_ fiber has lower PL intensity compared to the MoS_2_/PVDF fiber, which was attributed to the core-shell heterostructure, indicating that the recombination of photo-generated electron-holes was suppressed effectively. Therefore, the TiO_2_/PVDF@MoS_2_ could effectively enhance the separation of photo-generated charge carriers and extend the lifetime of photo-generated electron-hole pairs, leading to the superior photocatalytic activity.

### 3.4. Photocatalytic Performances

**Figure 9:**
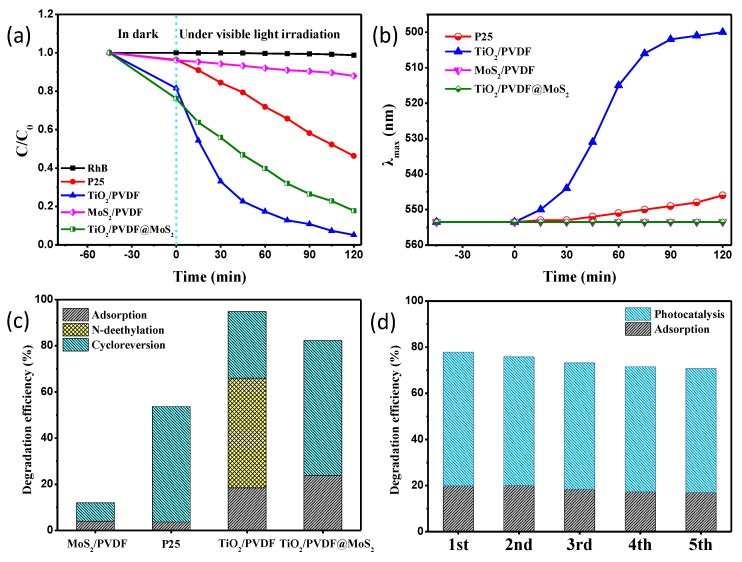
(**a**) Photocatalytic degradation curves of RhB over the samples: RhB without photocatalyst, P25, TiO_2_/PVDF, MoS_2_/PVDF and TiO_2_/PVDF@MoS_2_. (**b**) The wavelength of maximum absorption λ_max_ vs irradiation time. (**c**) The adsorption of RhB in dark as well as the *N*-deethylation and cycloreversion of RhB under visible light irradiation for different samples. (**d**) The degradation performance during 45-min adsorption of RhB in dark and 120-min photocatalytic degradation of RhB with the TiO_2_/PVDF@MoS_2_ core-shell heterostructured fibers. The experiment was repeated five times.

The photocatalytic performances of the as-prepared samples have been investigated by monitoring the time-dependent absorbance changes of RhB under visible light irradiation by using a white LED lamp. Figure 9a shows the time-dependent absorbance changes of RhB at 554 nm for RhB without photocatalyst, P25, TiO_2_/PVDF, MoS_2_/PVDF and TiO_2_/PVDF@MoS_2_, respectively. As can be seen, the absorbance of RhB without photocatalyst was almost unchanged under visible light illumination for 120 min, indicating that RhB was stable under visible light. Compared with the MoS_2_/PVDF reaction system, the TiO_2_/PVDF@MoS_2_ reaction system exhibited a superior photocatalytic efficiency, which was attributed to the core-shell heterostructure. Surprisingly, the P25 and TiO_2_/PVDF reaction systems both had good catalytic efficiency since they can only absorb UV light, as discussed in the UV-Vis section. Specifically, the TiO_2_/PVDF reaction system seemed to have the highest catalytic efficiency. There were two reasons for the photocatalytic effect under visible light irradiation for P25 and TiO_2_/PVDF reaction systems. On the one hand, the chromophore of RhB absorbed visible light and came to be in an excited state. Then the fast electron transferred from the excited chromophores to the conduction band of TiO_2_ led to degrade the RhB [52]. On the other hand, there were some oxygen vacancies related to a defect in P25 and TiO_2_/PVDF, as discussed in the PL spectra section, which could absorb visible light and lead to the degradation of RhB. 

By carefully observing the changes of the UV-Vis spectra at different times of the resultant samples, it was easy to find that the maximum absorption peaks of RhB solution for MoS_2_/PVDF and TiO_2_/PVDF@ MoS_2_ only had intensity changes rather than peak shift, as shown in Figure S5 (SI). While the maximum absorption peaks of RhB solution for P25 and TiO_2_/PVDF had not only intensity changes, but also peak shift. By comparing the optical photographs of RhB solution at different times of TiO_2_/PVDF and TiO_2_/PVDF@ MoS_2_, it could be found that the RhB solution for TiO_2_/PVDF constantly faded and turned yellow with the increase of time, however, the corresponding RhB solution for TiO_2_/PVDF@ MoS_2_ just faded, as displayed in Figure S6 (SI). 

The wavelength of maximum absorption λ_max_ vs irradiation time was shown in Figure 9b. It can be found that the maximum absorption λ_max_ for MoS_2_/PVDF and TiO_2_/PVDF@ MoS_2_ reaction system remained unchanged, while P25 and TiO_2_/PVDF shifted to short wavelength. There are two pathways to degrade RhB; one in the cleavage of chromospheres, and the other is the *N*-deethylation of RhB [53,54,55]. The products of RhB stepwise *N*-deethylation are *N*,*N*,*N*′-triethyl rhodamine (TER), *N*,*N*′-diethyl rhodamine (DER), *N*-ethyl rhodamine (MER) and rhodamine, respectively. The corresponding maximum absorption peaks for the products are located at 539, 522, 510, and 498 nm, respectively [56]. Therefore, part of RhB in the TiO_2_/PVDF reaction system eventually produced rhodamine through stepwise *N*-deethylation, while the RhB in P25 reaction system was only partial *N*-deethylation and did not form any of the above four final products. It can be calculated that 47.6% of the initial RhB molecules were transformed into rhodamine with *N*-deethylation by using the absorbance and molar extinction coefficient of RhB and rhodamine at 554 and 500 nm, respectively [57,58]. Figure 9c displays the three pathways in the MoS_2_/PVDF, P25, TiO_2_/PVDF and TiO_2_/PVDF@MoS_2_ reaction systems, adsorption, *N*-deethylation, and cycloreversion, respectively. As can be seen, TiO_2_/PVDF@MoS_2_ was more adsorptive than TiO_2_/PVDF, owing to the larger specific surface area. Benefitting from high photogenerated electron-hole separation resulting from the core-shell heterostructure, the amount of cleavage of RhB in the TiO_2_/PVDF@MoS_2_ reaction system was about 58.4%, more than 8.0% of the MoS_2_/PVDF reaction system, 50% of P25 reaction system, and 28.9% of TiO_2_/PVDF reaction system, respectively. Although the TiO_2_/PVDF reaction system showed a 76.5% degradation rate in Figure 9a, 47.6% degraded, RhB was only converted to a smaller rhodamine molecule.

In order to test the reusability of TiO_2_/PVDF@MoS_2_ core-shell heterostructured fibers, the recycled experiments were performed by using TiO_2_/PVDF@MoS_2_ core-shell heterostructured fibers five times and depicted in Figure 9d. Obviously, the TiO_2_/PVDF@MoS_2_ core-shell heterostructured fibers still maintained high adsorption and photocatalytic properties after repeated use five times. Besides, it can be seen that the adsorption and photocatalytic properties of the TiO_2_/PVDF@MoS_2_ core-shell heterostructured fibers decreased slightly with the increase of the reuse times, which was attributed to the loss of the photocatalyst on the fibers surface in the rinsing process in recovery.

The flexible characteristic was of great practical significance in recycling and reuses processes for photocatalytic materials. For the powdery photocatalysts, an inevitable problem was the loss of the photocatalyst during the separation process, leading to difficulties in recycle and reuse processes. In addition, some fibrous photocatalysts prepared by electrospinning generally required a high temperature to remove the organic components in the fibers, which resulted in very brittle fibrous photocatalysts. When these brittle fibrous photocatalysts were used in the photocatalytic experiments, the photocatalysts would be broken and lost due to being stirred, and thus couldn’t be recycled and reused. 

For the flexible TiO_2_/PVDF@MoS_2_ core-shell heterostructured fibers synthesized in this work, the presence of PVDF and a low temperature hydrothermal synthesis could keep it very flexible. Therefore, after five repeated uses, the photocatalytic performance of the flexible TiO_2_/PVDF@MoS_2_ core-shell heterostructured fibers was only slightly reduced due to the shedding of a few photocatalysts. In addition, the separation of the flexible TiO_2_/PVDF@MoS_2_ core-shell heterostructured fibers from the reaction system was very simple. It only needed to be clipped directly from the reaction system, which had a very important practical application value.

**Figure 10:**
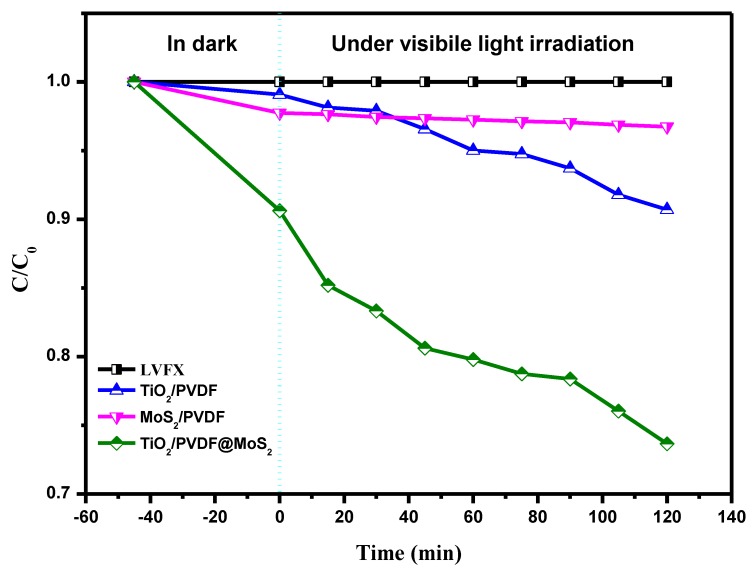
Photocatalytic degradation curves of LVFX over the samples: LVFX without photocatalyst, TiO_2_/PVDF, MoS_2_/PVDF and TiO_2_/PVDF@MoS_2_.

In order to further investigate the photocatalytic performance of the as-prepared samples, the photocatalytic degradation of LVFX which only absorbs UV light was carried out, as displayed in Figure 10. As can be seen, the absorbance of LVFX without photocatalyst remained unchanged under white light LED illumination for 120 min. Obviously, the as-obtained TiO_2_/PVDF@MoS_2_ core-shell heterostructured fibers still had the best photocatalytic properties in three kinds of photocatalysts, which was attributed to relatively large adsorption and high photogenerated electron-hole separation owing to the core-shell heterostructure. Meanwhile, MoS_2_/PVDF showed a very weak photocatalytic performance, except for the relatively large adsorption capacity. Surprisingly, TiO_2_/PVDF exhibited better photocatalytic performance than MoS_2_/PVDF under visible light irradiation. Taking into account the band-gap of TiO_2_/PVDF fibers and the spectrum of LED white light used in the experiment, the photocatalytic activity of TiO_2_/PVDF fibers membrane on LVFX was mainly attributed to the absorption of visible light by defects in TiO_2_/ PVDF fibers discussed earlier.

### 3.5. Photocatalytic Mechanism

Generally, h^+^, OH^•^ and $O_{2}^{-•}$ were recognized as the primary active species in the photocatalytic reaction [59]. To investigate the photocatalytic mechanism of the TiO_2_/PVDF and TiO_2_/PVDF@MoS_2_ fibers, control experiments with addition of individual scavengers for h^+^, OH^•^ and $O_{2}^{-•}$ were conducted on the photodegradation of RhB. 

**Figure 11:**
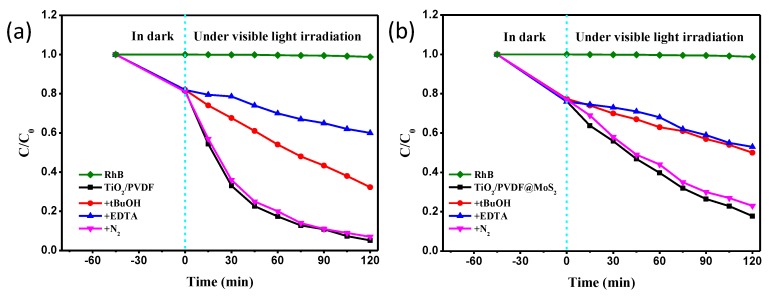
Control experiments with radical scavengers. (**a**) The relative concentration variation plots of RhB solution using TiO_2_/PVDF fibers as the photocatalyst. (**b**) The relative concentration variation plots of RhB solution using TiO_2_/PVDF@MoS_2_ fibers as the photocatalyst.

As shown in Figure 11a, the degradation of RhB was suppressed slightly by adding the N_2_ ($O_{2}^{-•}$ scavenger) to the TiO_2_/PVDF reaction system, indicating the $O_{2}^{-•}$ radical species, not main active oxidizing species in the photocatalytic process. However, it was significantly suppressed when the EDTA (h^+^ scavenger) was added, and a moderate suppressed degradation of RhB was appeared by the addition of tBuOH (OH^•^ scavenger). This result indicates that the h^+^ and OH^•^ were the main active oxidizing species involved in the TiO_2_/PVDF reaction system, with an order of h^+^ > OH^•^, during the photodegradation process. 

As mentioned above, the visible light photocatalytic ability of TiO_2_/PVDF fibers mainly came from oxygen vacancy related defects. Under visible light irradiation, electrons were excited from the valence band (VB) of TiO_2_ and trapped by defects, forming holes in the VB. The photo-generated holes migrated to the surface of the TiO_2_/PVDF fibers. On the one hand, parts of photo-generated holes were directly involved in oxidizing organic contaminants. On the other hand, parts of photo-generated holes reacted with water molecules to form OH^•^ [26]. Therefore, the addition of EDTA not only inhibits the h^+^ from participating in the oxidation of organic contaminants, but also inhibits the transformation of h^+^ into OH^•^. The addition of tBuOH prefers to inhibit OH^•^ to participate in the oxidation reaction rather than inhibit the participation of h^+^ in the oxidation process. Therefore, the addition of EDTA had a greater effect on the photodegradation of RhB than that of the addition of tBuOH. As described above, the photo-generated electrons in the TiO_2_ were trapped by the defects, so that very few photo-generated electrons migrated to the surface to react with the oxygen molecules in the reaction system to form $O_{2}^{-•}$. Therefore, the addition of N_2_ had little effect on photodegradation of RhB.

The photodegradation of RhB in the TiO_2_/PVDF@MoS_2_ reaction system with the addition of scavenger was displayed in Figure 11b. Similar to the TiO_2_/PVDF reaction system, the addition of EDTA had a stronger suppression on photodegradation of RhB than that of the addition of tBuOH and N_2_ in the TiO_2_/PVDF@MoS_2_ reaction system. Slightly different was that the inhibition of photodegradation of RhB with the addition of tBuOH in the TiO_2_/PVDF@MoS_2_ reaction system was very close to that of the addition of EDTA.

**Figure 12:**
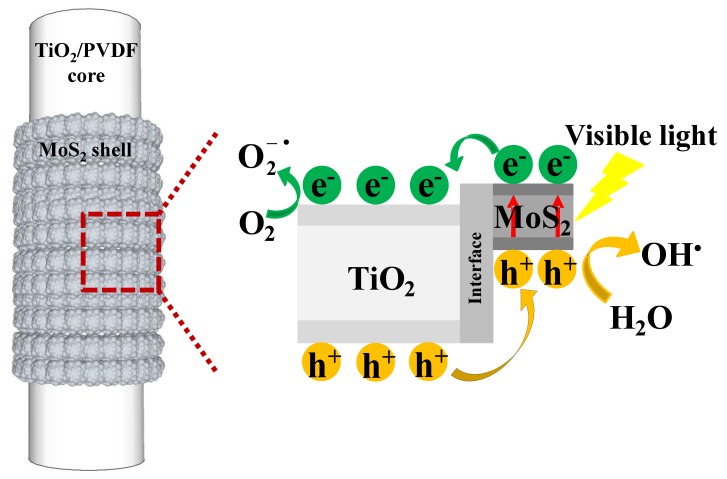
Schematic illustration for the photo-generated electron-hole separation and transfer process between TiO_2_/PVDF core and MoS_2_ shell.

To further understand this result, a schematic illustration for the photo-induced electron-hole separation and transfer process between TiO_2_/PVDF core and MoS_2_ shell is shown in Figure 12. Under visible light irradiation, electrons were excited from the valence band of MoS_2_ to its conduction band (CB), leaving a hole in the valence band. Since the VB of TiO_2_ was lower than that of MoS_2_, the photo-generated electrons were transferred from the CB of MoS_2_ to the CB of TiO_2_. [60] Accordingly, the holes migrated from the VB of TiO_2_ to the VB of MoS_2_. As a consequence, the photo-generated charge carriers were separated at the core-shell interface of the TiO_2_/PVDF@MoS_2_ heterostructure, as illustrated in Figure 12. As the h^+^ transported to the VB of MoS_2_ from the VB of TiO_2_, most of the h^+^ oxidized the water molecules in the solution to form OH^•^ [14] due to the relatively large specific surface area of TiO_2_/PVDF@MoS_2_ fiber compared to TiO_2_/PVDF fiber. On the other hand, a small portion of h^+^ was directly involved in the oxidation of organic contaminants. Hence, a close inhibitory effect on the photodegradation of RhB appeared in the addition of tBuOH compared the addition of EDTA. Meanwhile, the photo-generated electrons were transported to the CB of TiO_2_. Since TiO_2_ was in the core of the composite, only a very small number of electrons reacted with oxygen molecules in solution to form $O_{2}^{-•}$ [61] which could participate in the reaction of oxidizing organic contaminants. Therefore, the addition of N_2_ had little effect on inhibiting the photodegradation of RhB.

### 3.6. Self-Cleaning Performance

**Figure 13:**
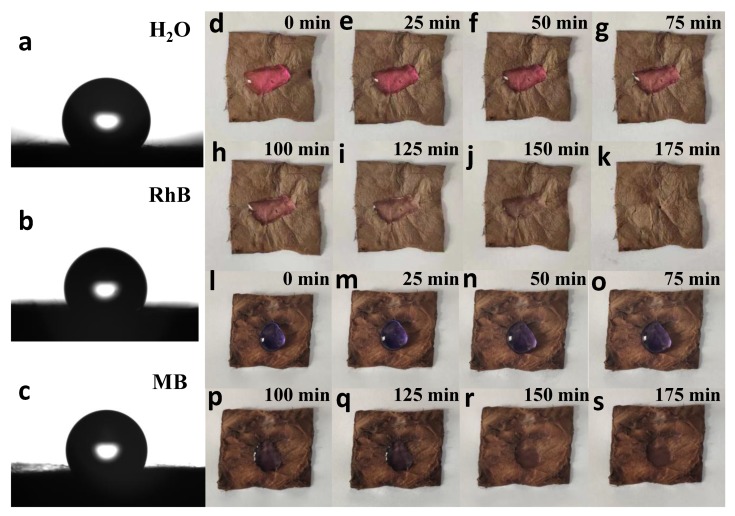
The optical images taken while the water and dye droplets come into contact to the surface of TiO_2_/PVDF@MoS_2_ core-shell heterostructured fibers (**a**–**c**). Photographs of the RhB (**d**–**k**) and MB (**l**–**s**) droplet on the surface of TiO_2_/PVDF@MoS_2_ core-shell heterostructured fibers under visible light illumination.

The wettability of the surface determines the main cleaning mechanism. One of the main parameters to characterize the wetting behavior of the surface is the static contact angle, which is the observable angle between liquid and solid. The contact angles of H_2_O, RhB, MB are 128.28°, 120.30°, 129.27°, respectively, as shown in the Figure 13a–c. Therefore, it can be obtained that the prepared TiO_2_/PVDF@MoS_2_ core-shell heterostructured fibers are hydrophobic to water and three dyes. 

In addition, the self-cleaning properties of TiO_2_/PVDF@MoS_2_ core-shell heterostructured fibers were tested by dropping 10 mg·L^−1^ of RhB and MB onto the surface of the as-prepared product under visible light illumination, as depicted in Figure 13d–s. It can be easily found that the colors of these two dyes almost disappear in about 150 min, meaning a good self-cleaning performance to these two dyes.

Furthermore, due to the hydrophobicity of TiO_2_/PVDF@MoS_2_ core-shell heterostructured fibers surface, water droplets can remain on the surface of the sample. Therefore, the dust can be removed from the sample surface by rolling the water droplets on the sample surface to achieve self-cleaning effect. As displayed in Figure S7 (SI), before dropping water onto the surface of the sample, a layer of dust is sprayed on the sample surface. Then, the droplets are then dropped onto the surface of the sample. Slightly tilted sample, droplets roll on its surface and bring dust down to reveal the original surface of the sample. This means that in the actual use, the as-prepared products can remove the adhered dyes or dust by sunlight or rainwater themselves, so as to reduce maintenance costs.

## 4. Conclusions

Novel flexible, recyclable, and reusable TiO_2_/PVDF@MoS_2_ fibers with core-shell heterostructures were synthesized by one-step low temperature hydrothermal method on the basis of TBOT/PVDF fibers prepared by electrospinning. The as-prepared core-shell heterostructured TiO_2_/PVDF@MoS_2_ fibers have good visible light absorption performance owing to the addition of narrow bandgap MoS_2_. As the core-shell heterostructure can greatly improve the photo-generated electron-hole pair’s separation efficiency, the as-prepared core-shell heterostructured TiO_2_/PVDF@MoS_2_ fibers have a very high dye contamination and antibiotic removal rates compared to the TiO_2_/PVDF fibers, under white light LED irradiation. Different from the main path way that the *N*-deethylation accompanied by cycloreversion to degrade RhB with TiO_2_/PVDF fibers, the path way to remove RhB with TiO_2_/PVDF@MoS_2_ core-shell heterostructured fibers is mainly the cleavage effect on chromospheres ring, which is a more complete degradation. Furthermore, the h^+^ was recognized as the most active species in the oxidation of organic pollutants. The results show that the as-prepared product has good photocatalytic activity and self-cleaning performance under visible light. Due to the presence of the PVDF polymer inside TiO_2_/PVDF@MoS_2_ the fiber membrane, the TiO_2_/PVDF@MoS_2_ core-shell heterostructured fiber has good flexibility and reusability, making itself a prospective material in the field of environmental management.

## Figures and Tables

**Table 1:** The absorption edge and energy band gap for the typical samples.

Typical Sample	Absorption Edge (nm)	Energy Band Gap (eV)
MoS_2_/PVDF	780.4	1.6
TiO_2_/PVDF@ MoS_2_	639.3	1.9
P25	375.7	3.3
TiO_2_/PVDF	361.8	3.4
TBOT/PVDF	335.5	3.7

## Supplementary Materials

The following are available online at https://www.mdpi.com/2079-4991/9/3/431/s1, Figure S1: The spectrum of LED white light used in the experiment, Figure S2: XRD patterns of (a) PVDF fibers, (b) MoS_2_/PVDF fibers & (c) MoS_2_ powder, Figure S3: EDS spectrum of TiO_2_/PVDF@MoS_2_ fiber, Figure S4: XPS survey spectrum of TiO_2_/PVDF@MoS_2_ fiber, Figure S5: Absorption spectra of RhB solutions at various irradiation times for different samples: (a) MoS_2_/PVDF, (b) P25, (c) TiO_2_/PVDF & (d) TiO_2_/PVDF@MoS_2_, Figure S6: Optical photographs of RhB solutions at various irradiation times for (a) TiO_2_/PVDF and (b) TiO_2_/PVDF@MoS_2_, Figure S7: The effect of a water drop rolling on the surface of the TiO_2_/PVDF@MoS_2_ core-shell heterostructured fibers to remove dust.
