# Supplementary material for: One-Step Low Temperature Hydrothermal Synthesis of Flexible TiO2/PVDF@MoS2 Core-Shell Heterostructured Fibers for Visible-Light-Driven Photocatalysis and Self-Cleaning
Source: Nanomaterials (Basel). 2019 Mar 14;9(3):431. doi: 10.3390/nano9030431 (PMC6473952; doi:10.3390/nano9030431)
Supplement: Supplementary file 1 [file nanomaterials-09-00431-s001.pdf]

Supporting Information (SI)

One-step Low Temperature Hydrothermal Synthesis of Flexible TiO_2/PVDF@MoS_2 Core-shell Heterostructured Fibers for Visible-Light-Driven Photocatalysis and Self-cleaning

Zhi-Guang Zhang [1,2], Hui Liu [1], Xiao-Xiong Wang [1], Jun Zhang [1], Miao Yu [1,3], Seeram Ramakrishna [4] and Yun-Ze Long [1,*]

[1] Collaborative Innovation Center for Nanomaterials & Devices, College of Physics, Qingdao University, Qingdao 266071, China; zhangzhiguangphysics@126.com (Z.-G.Z.); lhqddx@163.com (H.L.); wangxiaoxiong69@163.com (X.-X.W.); iamjunzhang@163.com (J.Z.); my2373@columbia.edu (M.Y.)

[2] College of Science & Information, Qingdao Agricultural University, Qingdao 266109, China

[3] Department of Mechanical Engineering, Columbia University, New York 10027, USA

[4] Center for Nanofibers & Nanotechnology, Nanoscience & Nanotechnology Initiative, Faculty of Engineering, National University of Singapore, Singapore 117576, Singapore; seeram@nus.edu.sg

* Correspondence: yunze.long@163.com or yunze.long@qdu.edu.cn; Tel: +86-139-5329-0681; Fax: +86-532-8595-5977

Figure Captions

Fig. S1 The spectrum of LED white light used in the experiment.

Fig. S2 XRD patterns of (a) PVDF fibers, (b) MoS_2/PVDF fibers & (c) MoS_2 powder.

Fig. S3 EDS spectrum of TiO_2/PVDF@MoS_2 fiber.

Fig. S4 XPS survey spectrum of TiO_2/PVDF@MoS_2 fiber.

Fig. S5 Absorption spectra of RhB solutions at various irradiation times for different samples: (a) P25, (b) TiO_2/PVDF, (c) MoS_2/PVDF & (d) TiO_2/PVDF@MoS_2.

Fig. S6 Optical photographs of RhB solutions at various irradiation times for (a) TiO_2/PVDF and (b) TiO_2/PVDF@MoS_2.

Fig. S7 The effect of a water drop rolling on the surface of the TiO_2/PVDF@MoS_2 core-shell heterostructured fibers to remove dust.

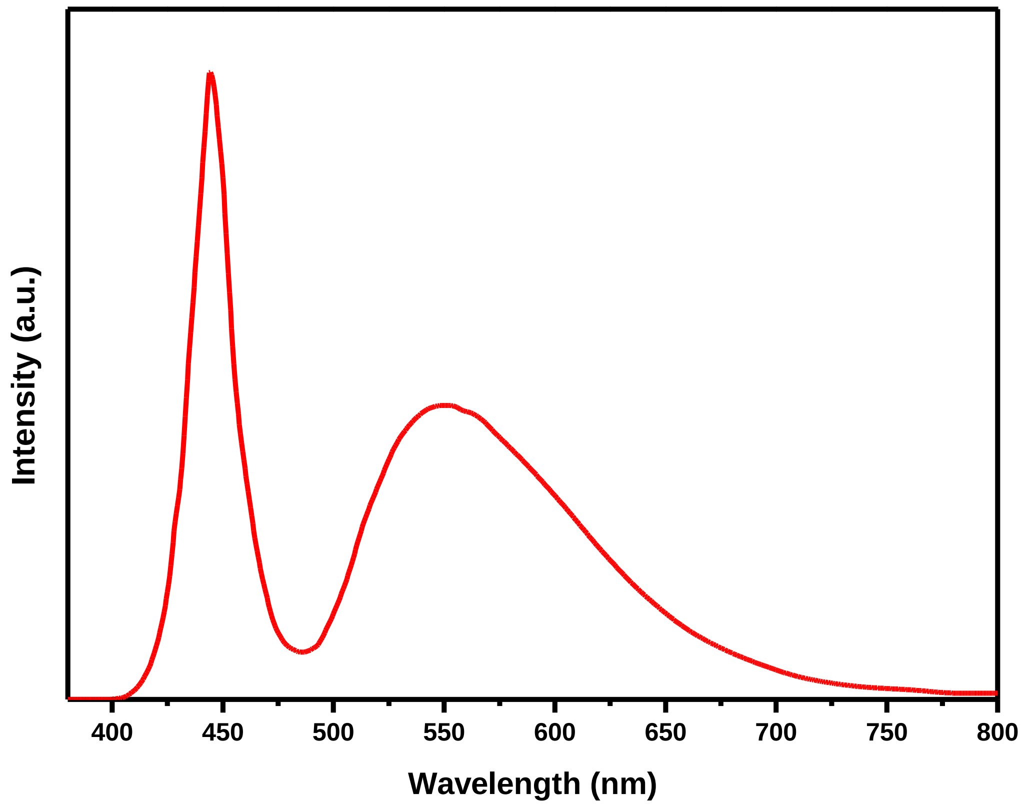

Fig. S1 The spectrum of LED white light used in the experiment.

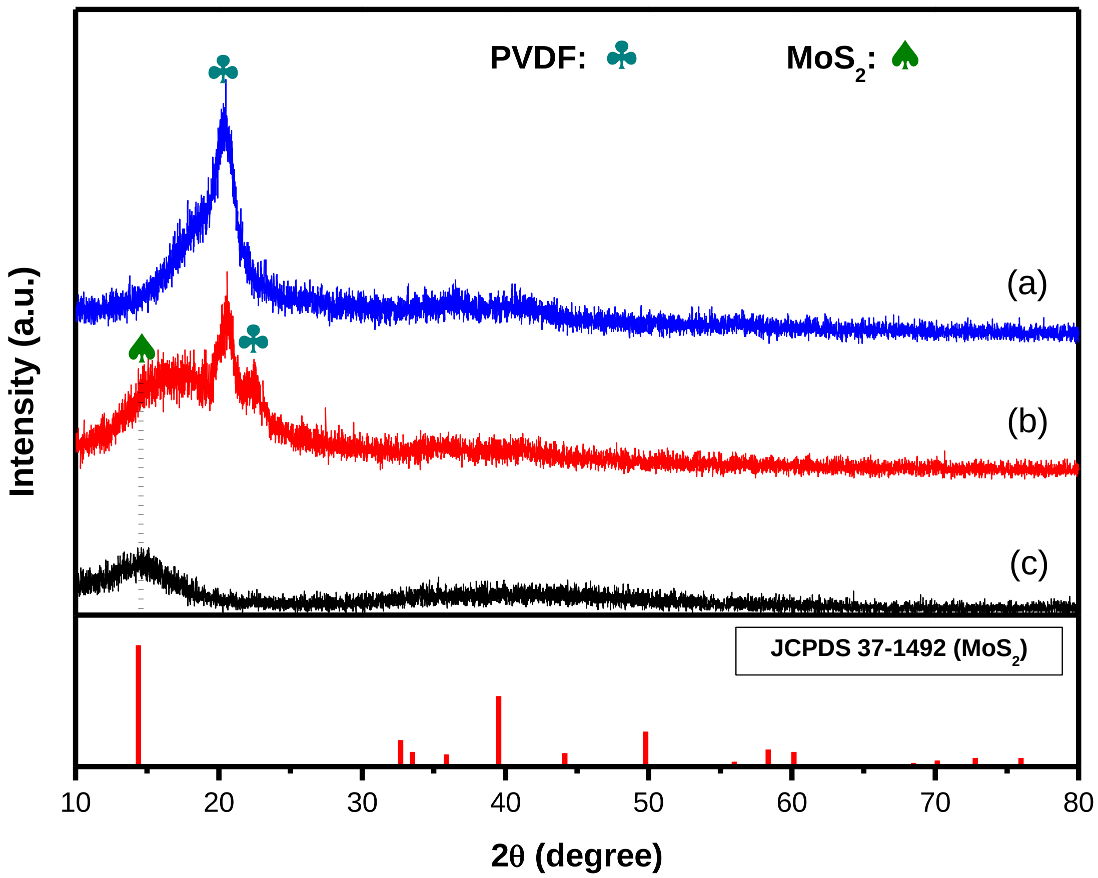

Fig. S2 XRD patterns of (a) PVDF fibers, (b) MoS_2/PVDF fibers & (c) MoS_2 powder.

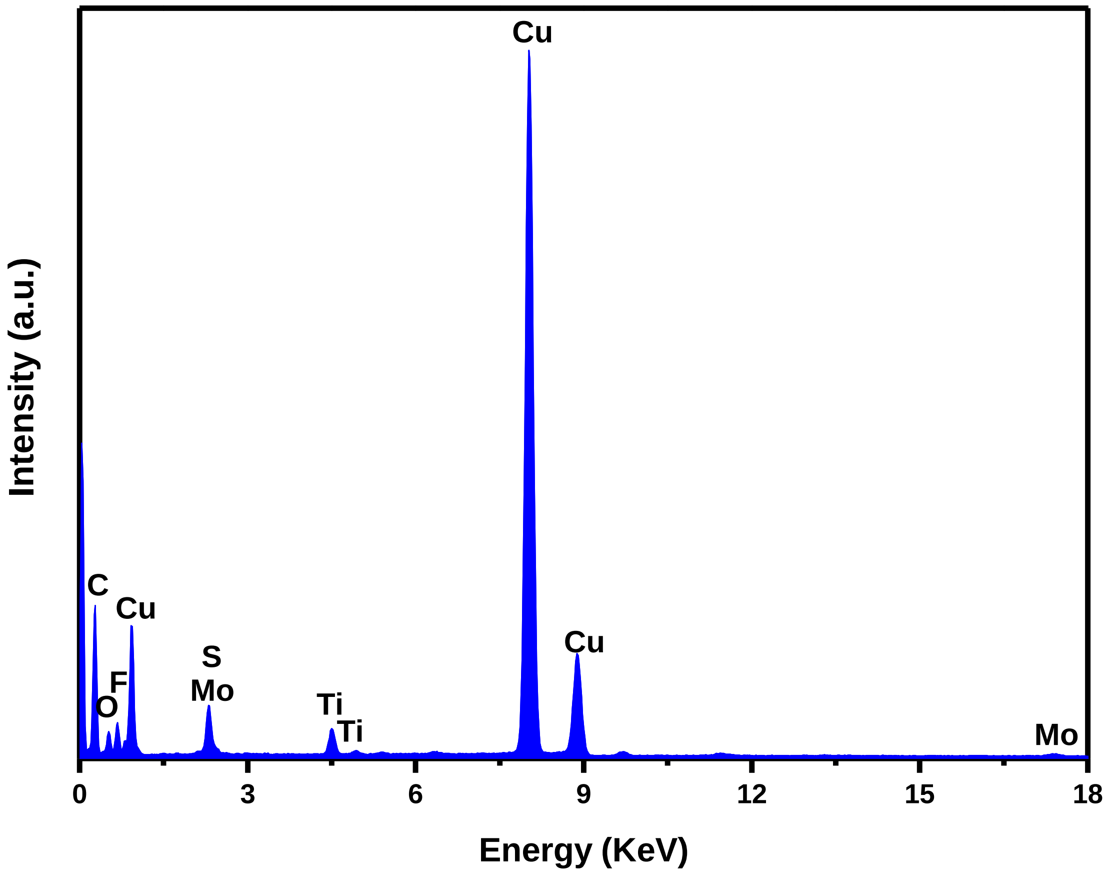

Fig. S3 EDS spectrum of TiO_2/PVDF@MoS_2 fiber.

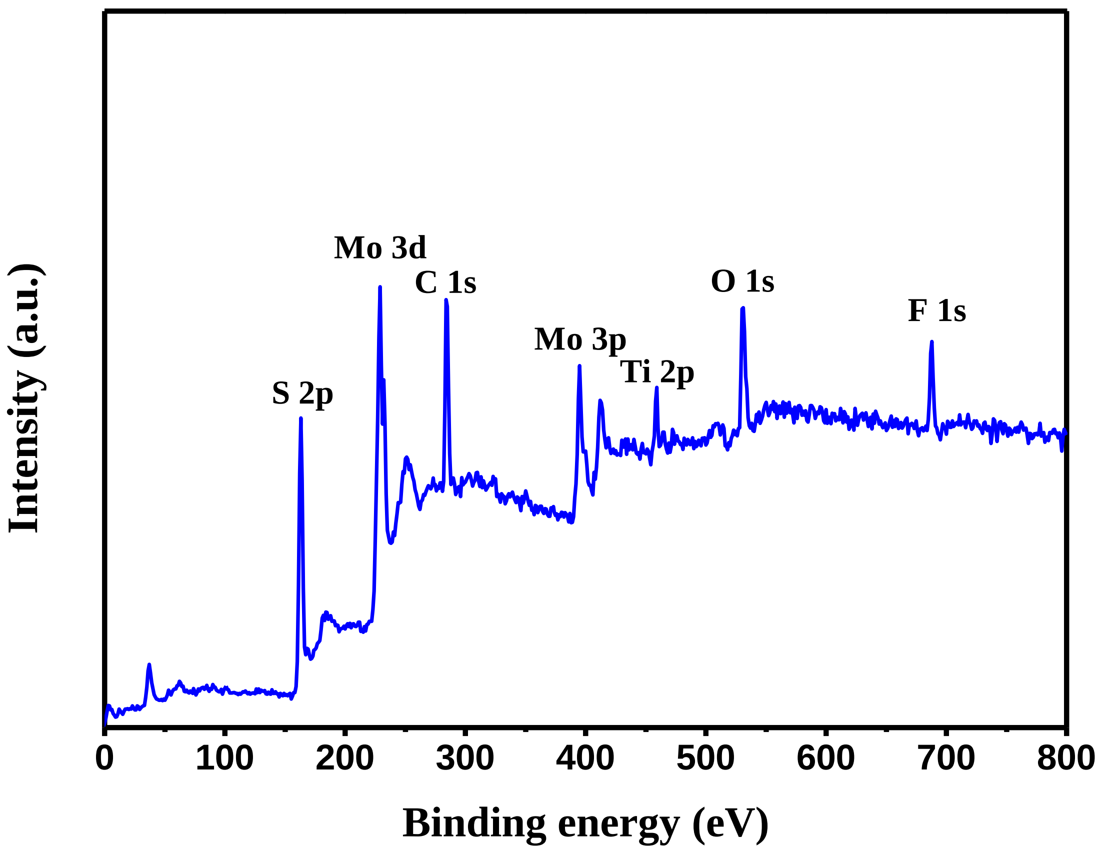

Fig. S4 XPS survey spectrum of TiO_2/PVDF@MoS_2 fiber.

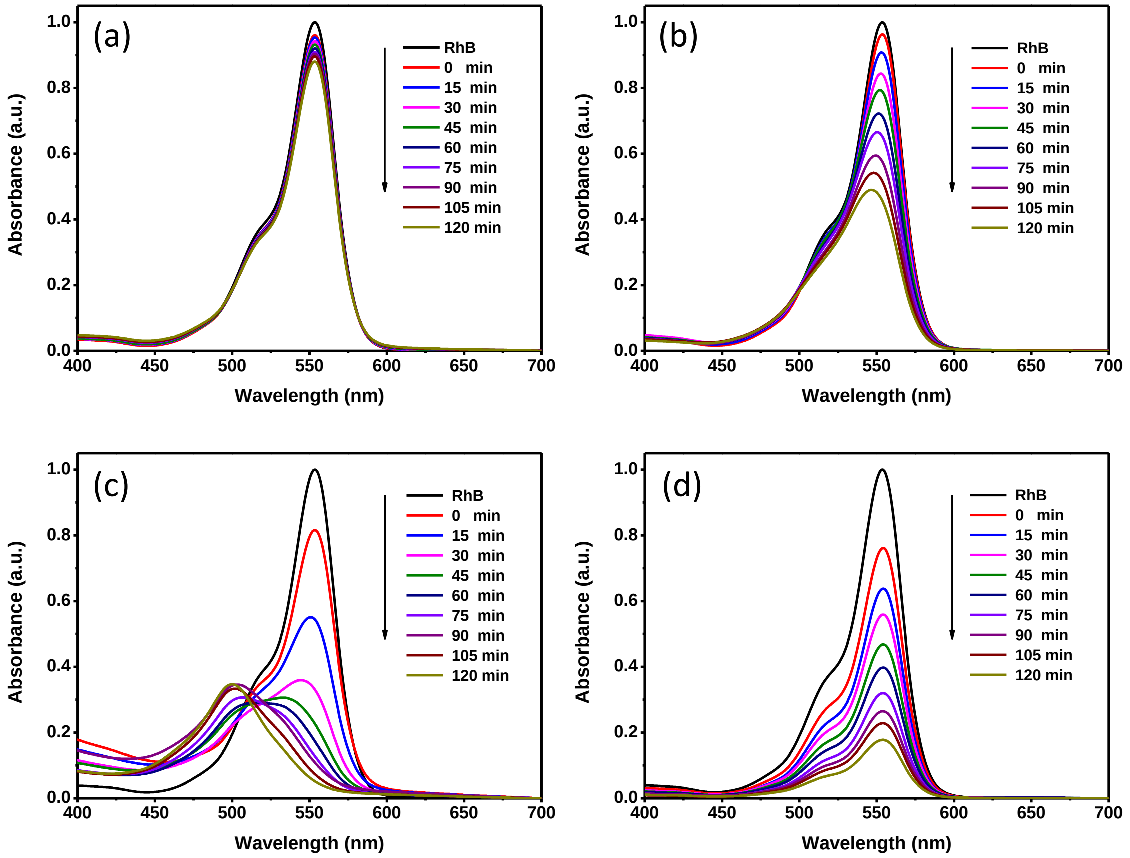

Fig. S5 Absorption spectra of RhB solutions at various irradiation times for different samples: (a) MoS_2/PVDF, (b) P25, (c) TiO_2/PVDF & (d) TiO_2/PVDF@MoS_2.

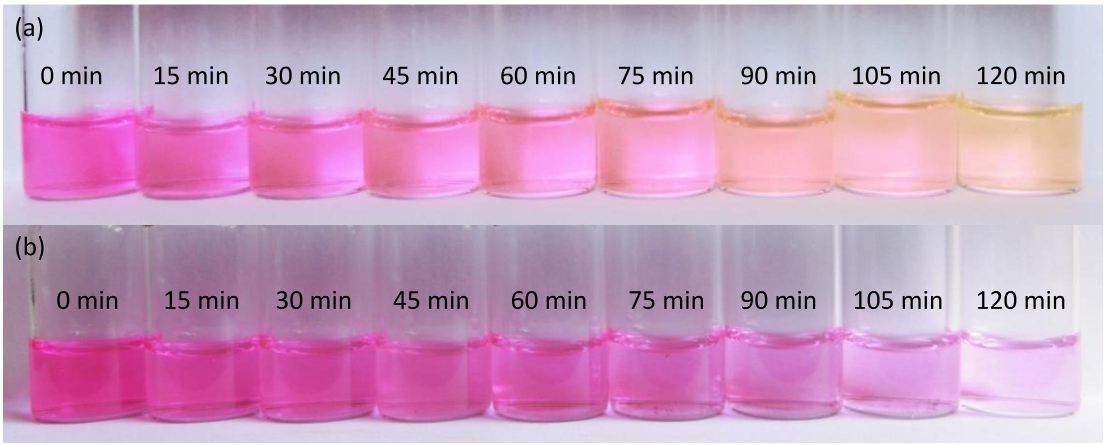

Fig. S6 Optical photographs of RhB solutions at various irradiation times for (a) TiO_2/PVDF and (b) TiO_2/PVDF@MoS_2.

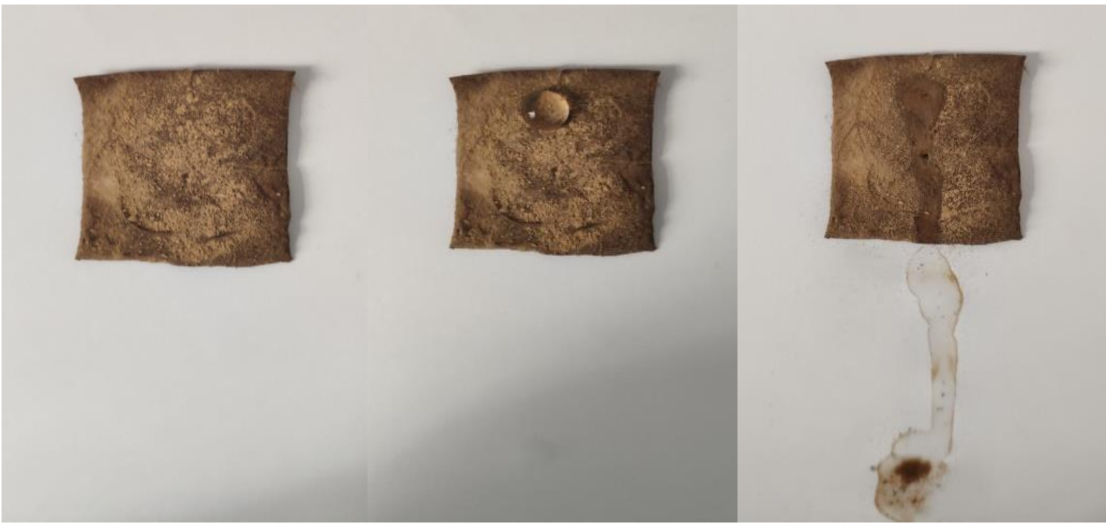

Fig. S7 The effect of a water drop rolling on the surface of the TiO_2/PVDF@MoS_2 core-shell heterostructured fibers to remove dust.
